# Methylation specific targeting of a chromatin remodeling complex from sponges to humans

**DOI:** 10.1038/srep40674

**Published:** 2017-01-17

**Authors:** Jason M. Cramer, Deborah Pohlmann, Fernando Gomez, Leslie Mark, Benjamin Kornegay, Chelsea Hall, Edhriz Siraliev-Perez, Ninad M. Walavalkar, M. Jeannette Sperlazza, Stephanie Bilinovich, Jeremy W. Prokop, April L. Hill, David C. Williams Jr.

**Affiliations:** 1 Department of Biochemistry and Molecular Biology, Virginia Commonwealth University, Richmond, VA, USA; 2 Department of Biology, University of Richmond, Richmond, VA, USA; 3 Department of Biochemistry and Biophysics, University of North Carolina at Chapel Hill, Chapel Hill, NC, USA; 4 Department of Pathology and Laboratory Medicine, University of North Carolina at Chapel Hill, Chapel Hill, NC, USA; 5 Department of Chemistry, University of North Carolina at Chapel Hill, Chapel Hill, NC, USA; 6 HudsonAlpha Institute for Biotechnology, Huntsville, AL, USA

## Abstract

DNA cytosine methylation and methyl-cytosine binding domain (MBD) containing proteins are found throughout all vertebrate species studied to date. However, both the presence of DNA methylation and pattern of methylation varies among invertebrate species. Invertebrates generally have only a single MBD protein, MBD2/3, that does not always contain appropriate residues for selectively binding methylated DNA. Therefore, we sought to determine whether sponges, one of the most ancient extant metazoan lineages, possess an MBD2/3 capable of recognizing methylated DNA and recruiting the associated nucleosome remodeling and deacetylase (NuRD) complex. We find that *Ephydatia muelleri* has genes for each of the NuRD core components including an EmMBD2/3 that selectively binds methylated DNA. NMR analyses reveal a remarkably conserved binding mode, showing almost identical chemical shift changes between binding to methylated and unmethylated CpG dinucleotides. In addition, we find that EmMBD2/3 and EmGATAD2A/B proteins form a coiled-coil interaction known to be critical for the formation of NuRD. Finally, we show that knockdown of EmMBD2/3 expression disrupts normal cellular architecture and development of *E. muelleri*. These data support a model in which the MBD2/3 methylation-dependent functional role emerged with the earliest multicellular organisms and has been maintained to varying degrees across animal evolution.

DNA methylation can be found across all three branches of life, from single cell organisms to mammals. Yet the functional role appears to have changed or expanded across evolution, and in many cases remains enigmatic. Bacteria, plants, fungi and most animals methylate DNA while some yeast do not. In bacteria, DNA methylation can repress viral replication and DNA transposition and function in the restriction endonuclease system^1^. Recently, it has been shown that fungal DNA methylation is also important to silencing of transposable elements while also contributing to developmental processes^2^. Both animals and plants use DNA methylation to silence whole chromosomes and to developmentally regulate specific genes. DNA methylation in animals predominantly involves the addition of a methyl group to the C5 position of a cytosine base. DNA methyltransferase enzymes (DNMTs) methylate symmetrically related cytosines in a cytosine-guanosine double stranded dinucleotide (CpG methylation)^3^ as well as individual cytosines in CpA, CpT, and CpC contexts (non-CpG methylation)^4^. DNMT3 introduces *de novo* methylation of cytosines, while DNMT1 restores symmetric methylation of hemi-methylated CpGs after DNA duplication and cell division in mammals. Early studies in *C. elegans* and *D. melanogaster* suggested that invertebrates lack functional DNMTs and consequently do not methylate cytosines^5^. However this view has changed since many, if not most, other invertebrates are now known to have functional DNMT1 and DNMT3 orthologs^6^,^7^,^8^,^9^,^10^.

Not only is the presence of DNA methylation more consistent and prevalent within vertebrate organisms when compared to invertebrates, the pattern of methylation differs as well. Vertebrates globally methylate most CpG dinucleotides, with a selective lack of methylation in regions of increased CpG content (CpG islands). Methylation of CpG islands within promoters, either in a developmentally regulated manner or aberrantly during carcinogenesis, leads to silencing of the associated gene. In contrast, most invertebrates studied thus far methylate their genomes in a mosaic pattern with roughly equal proportions of methylated and unmethylated DNA. Furthermore, invertebrate DNA methylation occurs largely within gene bodies as opposed to inter-genic regions. DNA methylation of gene bodies is often associated with active transcription in both vertebrates and invertebrates^11^. Together these observations suggest that the role of DNA methylation changed or expanded with the emergence of vertebrates.

Our laboratory has been studying the structure and function of the methyl-cytosine binding domain (MBD) family of proteins^12^,^13^,^14^,^15^. These proteins share an approximately 60 amino acid domain that can selectively bind to symmetrically methylated CpG dinucleotides. While invertebrates have only a single MBD protein (MBD2/3)^6^,^9^,^16^, vertebrate genomes have duplicated the ancestral MBD2/3 gene to generate highly homologous MBD2 and MBD3 proteins, as well as expanded the family by associating the MBD with different regulatory (MeCP2 and MBD1) and enzymatic (MBD4) domains^17^. The MBD2 and MBD3 proteins each recruit the large nucleosome remodeling and deacetylase (NuRD) complex in a mutually exclusive manner^18^. Most MBD3 proteins lack a strong preference for methylated DNA (with the exception of *Xenopus* MBD3) while MBD2 proteins show 100-fold and higher methylation selectivity^19^,^20^,^21^. Therefore, the change in methylation pattern between invertebrate and vertebrate organisms coincides with duplication of the ancestral MBD2/3 protein to generate NuRD complexes with differing degrees of methylation selectivity. This concordance raises an important question - did the ancestral MBD2/3 protein initially function to target NuRD to methylated DNA, or did methylation selectivity and/or association with NuRD arise with the change in methylation pattern?

To address this fundamental question and better understand the evolving functional roles of DNA methylation in animals, we sought to investigate the structure and function of the MBD2/3 and NuRD proteins from sponges, arguably the most ancient extant metazoan lineage^22^. Recent whole genome sequencing of the marine demosponge, *Amphimedon queenslandica*, showed evidence for DNA methylation with a relative depletion of CpG dinucleotides, increase in CpT and CpA dinucleotides, and the presence of genes for the DNA methylation machinery^23^. We chose to study DNA methylation in a model freshwater sponge, *Ephydatia muelleri*, that can be grown in the laboratory from gemmule through five stages of development. We have established methods to knockdown gene expression in *E. muelleri* using double stranded RNA^24^ such that we can study MBD2/3-dependent gene expression and development in a controlled laboratory environment. Importantly, a draft *E. muelleri* transcriptome has recently become available allowing us to identify and clone the EmNuRD components.

In these studies, we find that *E. muelleri* has the appropriate genes to methylate DNA and form an MBD2-NuRD complex. We confirm the presence of DNA methylation across all stages of development of *E. muelleri* as sponges hatch and form the aquiferous system. Methylation coincides with expression of EmMBD2/3 which is also expressed at all developmental stages. Furthermore, we show that EmMBD2/3 selectively binds methylated DNA and forms a coiled-coil interaction critical to recruitment of NuRD. As a comparison, we tested DNA binding and coiled-coil complex formation for MBD2/3 from *Drosophila melanogaster,* an invertebrate that does not methylate CpG dinucleotides at high levels. The DmMBD2/3 does not bind DNA but does form the critical coiled-coil interaction, suggesting MBD2/3 contributes to NuRD complex formation independent of DNA methylation. Finally, we show that reducing the expression of EmMBD2/3 during *E. muelleri* development leads to abnormalities at the leading edge of sponge growth in the basal pinacoderm. These studies clearly demonstrate that methylation selectivity and function of the MBD2-NuRD complex likely emerged early in animal evolution in the sister group to all metazoans.

## Results and Discussion

### E. muelleri NuRD complex

**Figure 1:**
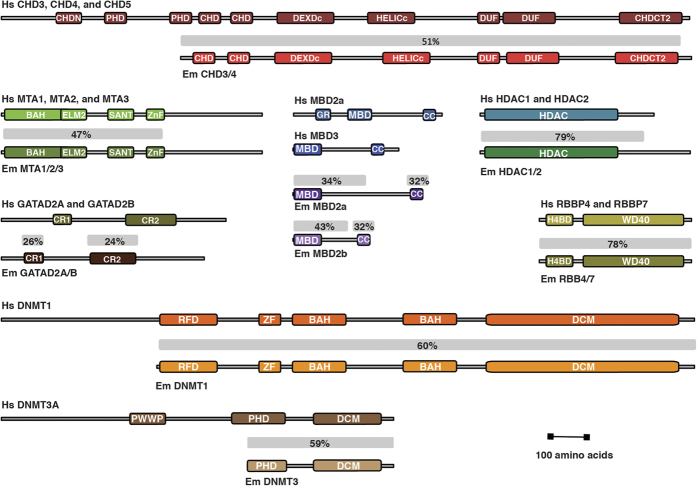
Conservation of NuRD core components. The domain architecture and conservation (% identity) is shown for each of the NuRD core components and DNA methyltransferase enzymes between human (Hs) and *E. muelleri* (Em) species.

We searched an *E. muelleri* transcriptome for homologs to each of the five NuRD core components. As shown in Fig. 1, *E. muelleri* contains a single paralog for each NuRD protein. The single domain HDAC1/2 and Rbbp4/7 proteins are highly similar to the human orthologs (79% and 78% identity, respectively) consistent with their conserved roles in multiple complexes that deacetylate histones. The identifiable functional domains of MTA1/2 (BAH, ELM, SANT, and Zn finger) are also well conserved, showing 47% identity across this region. However, the C-terminal region does not show sufficient similarity to align with a BLAST® search^25^. The functional domains of the large chromatin remodeling protein, CHD3/4, show 51% identity. Interestingly, though, EmCHD3/4 lacks the N-terminal PHD domains known to bind histone tails in the mammalian protein^26^,^27^. The *A. queenslandica* CHD3/4 orthologue appears to lack PHD domains as well, which suggests that these domains were added in other organisms after the demosponges split from the common ancestor. As more sponge genomes become available, this aspect of the data will become clearer.

In contrast, both GATAD2A/B and MBD2/3 orthologs show much less conservation, even within their functional domains. The GATAD2A/B protein contains two small conserved regions, CR1 and CR2^28^, which show only 26% and 24% identity with the human protein, respectively. The remaining regions are predicted to be largely unstructured in isolation and, as such, are poorly conserved. Similarly the MBD2/3 protein contains methyl-cytosine binding (MBD) and coiled-coil (CC) domains separated by an intrinsically disordered region^13^,^17^. We identified two isoforms of EmMBD2/3, which differ by large insertions within the intrinsically disordered region. These two isoforms show only 34% and 43% identity within the N-terminal half as compared to HsMBD2, while the coiled-coil domain shows 32% identity.

These findings indicate that each of the NuRD core components was likely present in the earliest multicellular animals. The level of conservation inversely correlates with inherent disorder in each of the proteins. The critical binding regions, even within GATAD2A/B and MBD2/3, are conserved. Hence *E. muelleri* contains all of the necessary components to form a functional NuRD complex.

### EmMBD2/3 selectively binds methylated DNA

We determined whether *E. muelleri* has methylated cytosine bases across developmental stages. We identified EmDNMT1 and EmDNMT3 orthologs which are responsible for maintenance and *de novo* methylation, respectively (Fig. 1). We first measured global cytosine methylation levels during *E. muelleri* development using a colorometric ELISA-based method (MethylFlash^TM^). Global DNA methylation was present across all stages of sponge development from gemmule hatching (Stage 0) to fully functioning juvenile adult (Stage 5), and this method indicated that methylation levels increased at stage five when the aquiferous system is formed (Table 1). Given that DNA methylation was detected during all stages of development, we also directly quantified both 5-methy-2′-deoxycytidine (5mdC) and 5-hydroxymethyl-2′-deoxycytidine (5HmdC) levels using SRM-based liquid chromatography mass spectrometry (Table 1). The range of 5mdC levels across *Ephydatia* developmental stages was between 4.38 and 5.39%. These levels are similar to those reported for several marine sponges^5^. 5HmdC levels were much lower and ranged from not detectable to 0.018%. The increase in methylation levels in stage 5 sponges that was observed using the ELISA-based method was not detected using mass spectrometry. Given that mass spectrometry is a more precise method, we do not find significant differences in global DNA methylation levels across *Ephydatia* developmental stages.

**Figure 2:**
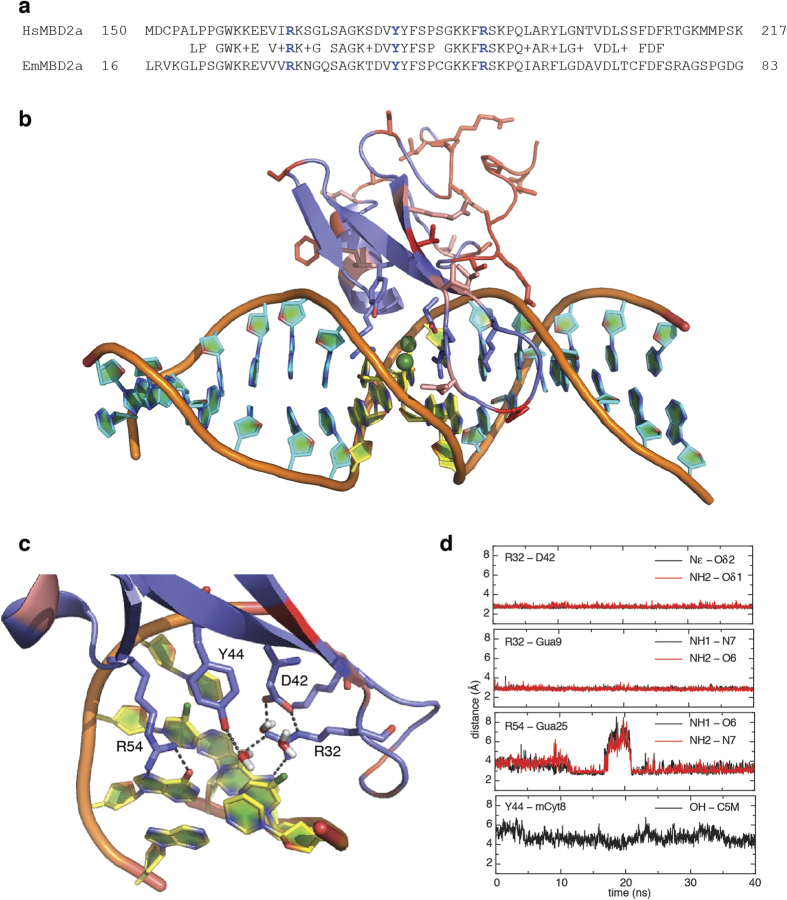
Structural conservation of the MBD of MBD2. (**a**) Alignment of the MBDs from EmMBD2/3 and HsMBD2 show a high degree of identity including residues critical for recognizing methylated cytosines (blue). (**b**) A cartoon diagram is shown of the final frame from a 40 ns molecular dynamics simulation of the MBD from EmMBD2. Residues that differ between HsMBD2 and EmMBD2/3 are shown as sticks in pink (conservative changes) and red (non-conservative changes) and residues critical for recognizing mCpG are shown as sticks in blue. The mCpG dinucleotide is shown in yellow with the methyl group highlighted as a green sphere. A close-up view (**c**) shows that the critical residues at the protein-DNA interface form the appropriate structure and hydrogen bonding network for methylation selectivity including several water molecules surrounding the methyl group of methyl-cytosine. (**d**) The distances between key hydrogen bond donor and acceptors and between the tyrosine hydroxyl and methyl carbon are plotted. These critical interactions are highly stable and maintained throughout the duration of the simulation.

Based on these observations, we tested whether the MBD of EmMBD2/3 preferentially binds methylated DNA. As described previously^12^,^29^, the MBDs specifically recognize CpGs through two highly conserved arginine residues. These arginines form bidentate hydrogen bonds with the symmetrically opposed guanosine bases of the double stranded dinucleotide. Selectivity for methylation derives from a highly conserved tyrosine residue whose sidechain hydroxyl group replaces a structured water molecule surrounding the C5 methyl group. Hence both HsMBD3, which has a phenylalanine instead of tyrosine, and MBD4, which reorients the tyrosine away from the DNA, show much lower selectivity for mCpG as compared to other MBDs. EmMBD2/3 contains all three critical residues (Fig. 2a) indicating it should selectively bind mCpG. We generated a model of the EmMBD2 MBD bound to a methylated DNA sequence based on the solution structure of *Gallus gallus* MBD2 (PDB ID 2ky8)^12^ and using MODELLER comparative modeling software^30^. We then carried out 40 ns of molecular dynamics simulation of the protein:DNA complex in explicit water containing 75 mM NaCl. A plot of the root mean square fluctuation (RMSF) calculated over a window of 10 steps shows that the complex stabilizes within the first the first 15 ns of simulation (Supplementary Fig. S1). As can be seen in Fig. 2b, when we model the domain based on the conserved MBD fold, the critical arginine and tyrosine residues can form the appropriate interactions that drive methylation selectivity. In particular, both arginine residues maintain stable bidentate hydrogen bonds with the symmetrically related guanosine bases and the tyrosine hydroxyl interacts with the shell of water surrounding the cytosine methyl group (Fig. 2c,d). While this simulation is relatively short and likely would not reveal large conformational rearragements, the results do indicate that the sequence differences as compared with vertebrate MBD2 do not overtly conflict with forming a stable interaction with DNA.

**Figure 3:**
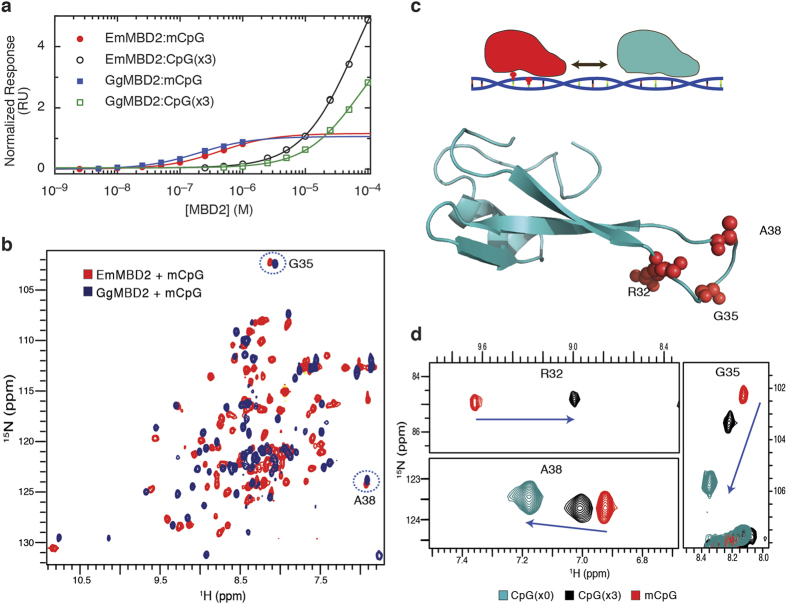
EmMBD2 selectively binds methylated DNA. (**a**) Surface plasmon resonance analyses shows that EmMBD2/3 binds with similar affinity and selectivity for methylated (mCpG) and unmethylated (CpG(x3)) DNA as GgMBD2. (**b**) 2D ^15^N-HSQC spectra show similar chemical shift distributions for EmMBD2/3 and GgMBD2 with very distinct resonances for reporter residues G35 and A38 (circled and labeled). (**c**) As we described previously^14^, the unusual chemical shifts for the sidechain of R32(^15^Nε^1^Hε) and the backbone amides of G35 and A38 show large differences between mCpG specific (red) and non-specific (cyan) binding modes. These three residues, shown as red spheres in the cartoon diagram of EmMBD2/3 MBD, are on a loop connecting the central β-strands of the MBD. R32 forms critical bidentate hydrogen bonds with a guanosine base in the CpG dinucleotide and with the sidechain of D42 (see Fig. 2) which stabilizes this loop. (**d**) 2D ^15^N-HSQC spectra show that resonances for these three residues in EmMBD2/3 show large, and proportional chemical shift changes whether bound to methylated (mCpG), unmethylated (CpG(x3)), or DNA that lacks any CpG dinucleotides (no CpG). This behavior is very similar to what we previously described for GgMBD2^14^ and indicates that EmMBD2/3 preferentially localizes to densely methylated regions.

We next tested selectivity by measuring binding affinity for a 17 base pair oligonucleotide with and without methylation of a centrally located CpG dinucleotide. As shown in Fig. 3a and Table 2, EmMBD2/3 binds with high affinity and selectivity (100–200 fold) for mCpG as compared to CpG, which is remarkably similar to HsMBD2.

We previously found that specific resonances in 2D ^15^N-HSQC spectra of MBD2 and MBD3 show large chemical changes when bound to methylated versus unmethylated DNA. Based on these chemical shift changes, we have shown that MBD2 exclusively binds to a methylated CpG when present, while MBD3 exchanges rapidly between CpG-specific and non-specific binding modes^14^. Hence, these chemical shifts reflect the degree of methylation selectivity and the distribution between CpG and non-specific binding modes. These same resonances in the ^15^N-HSQC spectrum of the EmMBD2/3 MBD show chemical shift changes remarkably similar to that of *G. gallus* MBD2 (Fig. 3b). Three reporter resonances for residues G35, A38, and R32 (Fig. 3c) show large and proportional changes between binding DNA without any CpGs, with three unmethylated CpGs, and with a single mCpG and two unmethylated CpGs. As we demonstrated previously, this linear and proportional change in chemical shift indicates that EmMBD2/3 exchanges rapidly between CpG specific and non-specific binding modes^14^. Furthermore, the chemical shifts of these reporter residues when bound to the methylated DNA indicate that the *E. muelleri* protein domain localizes almost exclusively to methylated sites when present.

Together, these findings establish that the methyl-cytosine binding domain of EmMBD2/3 biochemically recognizes methylated DNA in a manner remarkably similar to that of the vertebrate MBD2 ortholog and with very similar affinity and methylation selectivity.

### DmMBD2/3 does not bind DNA

**Figure 4:**
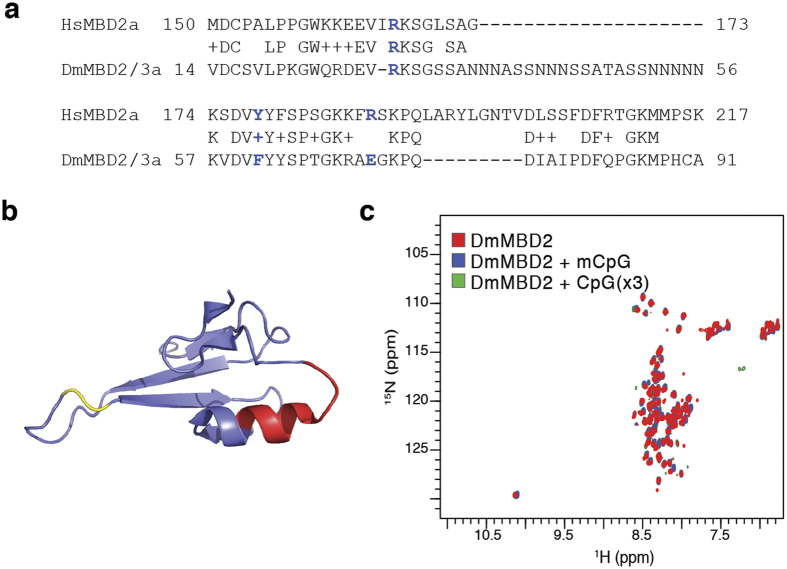
The DmMBD2/3 MBD does not bind DNA. (**a**) Alignment of the MBD from DmMBD2/3 with HsMBD2 reveals a large 20 amino acid glutamine rich insertion and a 9 amino acid deletion. In addition, two of the residues critical for binding methylated DNA (highlighted blue) have changed (F61 and E71). (**b**) A cartoon diagram of the MBD from GgMBD2 demonstrates that the large insertion occurs between residues highlighted in yellow in the loop connecting the central β-strands. The deletion, highlighted in red, eliminates much of the C-terminal α-helix and hydrophobic core of the domain. (**c**) 2D ^15^N-HSQC spectra of DmMBD2 MBD in isolation (red) or in the presence of methylated (blue) and unmethylated DNA (green) show characteristic features of an unstructured peptide without any evidence of binding DNA.

In contrast to *E. muelleri, D. melanogaster* lacks DNMT1/DNMT3 enzymes and does not appear to methylate CpG dinucleotides at high levels (although the presence of any methylation remains controversial)^31^,^32^,^33^. Yet *D. melanogaster* does possess orthologs for each of the NuRD components including DmMBD2/3. Comparing the MBDs from DmMBD2/3 and HsMBD2 reveals a large 20 amino acid glutamine rich insertion within the loop that extends down the major groove in the structure of the vertebrate ortholog^12^ as well as a 9 amino acid deletion involving most of the C-terminal α-helix (Fig. 4a,b). The differences include two of the critical residues involved in selectively binding methylated DNA (F61 and E71) and eliminate much of the hydrophobic core provided by the α-helix. To test whether DmMBD2/3 MBD binds DNA, we collected 2D ^15^N-HSQC spectra of the domain in isolation and in the presence of methylated and unmethylated DNA (Fig. 4c). The resulting spectra are characteristic of an unstructured domain with sharp peaks and very little dispersion in the ^1^H dimension. Furthermore, the presence of DNA does not induce any chemical shift changes to indicate binding or adopting a regular structure. These data demonstrate that, despite previous reports, the isolated MBD from DmMBD2/3 does not recognize DNA and is in fact an unstructured domain in isolation.

### EmMBD2/3 binds EmGATAD2A/B

We previously biophysically characterized and determined the structure of a coiled-coil complex formed between vertebrate MBD2 and GATAD2A (p66α), and showed that this interaction was critical for recruitment of CHD4 and methylation-dependent gene silencing by NuRD^34^,^35^. In the current studies we tested whether the invertebrate coiled-coiled domains from EmMBD2/3 and EmGATAD2A/B could form a similar stable coiled-coil complex.

**Figure 5:**
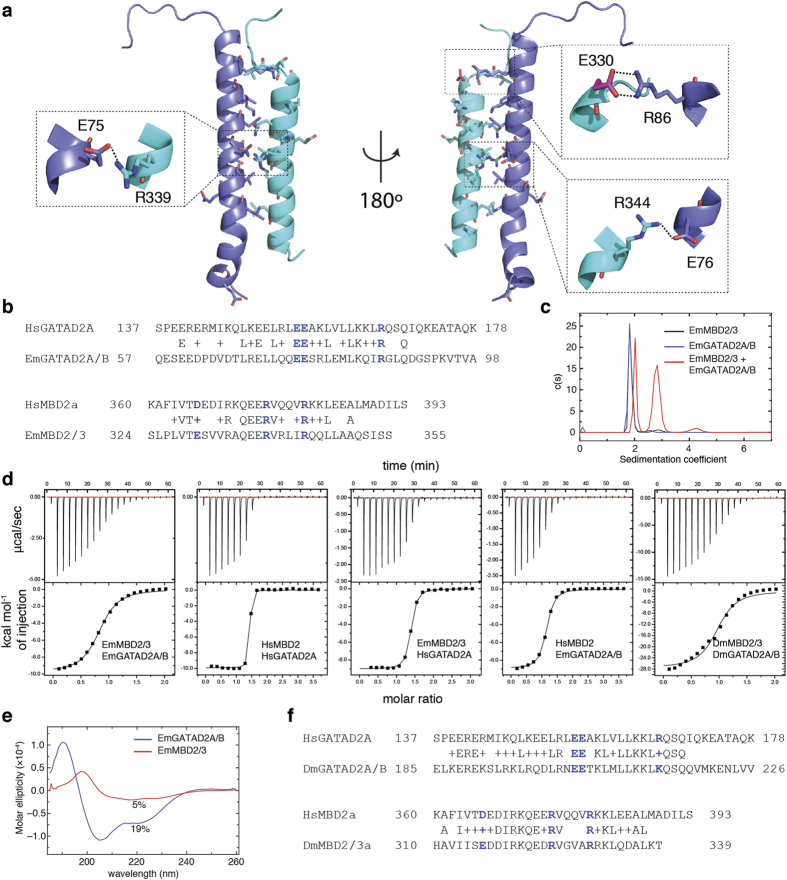
The coiled-coil interaction between EmMBD2/3 and EmGATAD2A/B. (**a**) A cartoon diagram depicts the final frame from a 40ns molecular dynamics simulation of a model of the coiled-coil interaction between EmMBD2/3 (cyan) and EmGATAD2A/B (blue). The amino acids residues conserved between the human and *E. muelleri* complexes (shown in sticks) are primarily located at the interface between the proteins. Expanded views (dashed boxes) show preservation of three critical ionic and hydrogen bond interactions, with the only difference between species involving a conservative aspartate to glutamate (E330) change (highlighted in magenta). (**b**) Alignment of the coiled-coil domains shows relatively low overall identity but with conservation of critical ionic (blue) and interface residues. (**c**) Analytical ultracentrifugation sedimentation velocity analyses indicates that the isolated EmMBD2/3 (black) and EmGATAD2A/B (blue) are largely monomeric while a mixture of the two (red) sediments as a combination of monomeric and heterodimeric species. (**d**) Isothermal titration calorimetry shows that the EmMBD2/3:EmGATAD2A/B and DmMBD2/3:DmGATAD2A/B complexes bind with relatively low affinity as compared to HsMBD2:HsGATAD2A; whereas cross-species (Em:Hs) complexes bind with intermediate affinities. (**e**) Circular dichroism measurements show that the EmGATAD2A/B (blue) an EmMBD2/3 (red) domains have relatively low helical content in isolation. (**f**) Alignment of DmMBD2/3 and DmGATAD2A/B with human orthologs shows a higher degree of conservation as compared to sponge.

We first built a model of EmMBD2/3:EmGATAD2A/B based on the NMR structure of the human coiled-coil complex (PDB ID 2l2l)^34^. The initial model was generated with MODELLER followed by 40 ns molecular dynamics simulation with explicit water. A plot of the RMSF shows that the coiled-coil complex remains stable throughout the simulation (Supplementary Fig. S1). In Fig. 5a, we show the final frame from this simulation with residues conserved between human and *E. muelleri* depicted in sticks. While the overall identity is fairly low (Fig. 5b), residues involved in direct contact between the two proteins are highly conserved. We previously identified three intermolecular ionic/hydrogen bond interactions critical for high affinity binding^34^,^35^. The equivalent interactions are maintained in the *E. muelleri* complex (Fig. 5a) with only one amino acid difference from a conservative aspartate to glutamate change (Fig. 5a). Although a relatively short simulation, these findings suggest that the two *E. muelleri* proteins can form the same critical coiled-coil interaction to form NuRD.

We then tested whether the two coiled-coil domains could bind in isolation. Gel filtration analysis shows that the dominant peak elutes earlier when the two proteins are mixed together than either of the individual peptides in isolation (Supplementary Fig. S2) consistent with complex formation. Analytical ultracentrifugation analyses of coiled-coil domains with thioredoxin fusion tags show that each peptide behaves largely as a single monomeric species in isolation (Fig. 5c) with measured (expected) molecular weights of 23.7 (21.3) kDa and 26.8 (22.7) kDa for EmMBD2/3 and EmGATAD2A/B coiled-coil domains, respectively. A 100 μM mixture of the peptides contains three species with sedimentation coefficients compatible with a mixture of monomers (27.6 kDa, 36.0%), heterodimeric (45.1 kDa, 58.1%), and multimeric complexes (83.3 kDa, 5.2%). Subsequent isothermal titration calorimetry analysis (Fig. 5d and Table 3) shows that the two peptides bind with relatively low affinity (K_D_ = 9,200 ± 700 nM). Given this low affinity, approximately 69% of the peptides will be in complex when the total concentration of each peptide is 100 μM. Hence the measured binding affinity agrees well with the observed mixture of monomer and dimer species by analytical ultracentrifugation.

In addition, we measured affinity for the human complex HsMBD2:HsGATAD2A (K_D_ = 48 ± 16 nM) as well as the cross-species complexes HsMBD2:EmGATAD2A/B (K_D_ = 1,300 ± 100 nM) and EmMBD2/3:HsGATAD2A (K_D_ = 380 ± 30 nM). The difference in affinity between human and sponge complexes reflects a reduction of approximately 2.8 kcal/mol in entropic penalty between the two complexes (Table 3). The cross-species complexes show binding affinities intermediate between that of the two native complexes. These results indicate that the *E. muelleri* domains likely interact in a similar manner, but that binding involves a large reduction in entropy.

We previously established that the helical propensity of the isolated HsMBD2 and HsGATAD2A coiled-coil domains correlates with the binding affinity. The ability to preform the coiled-coil domain helices allows for much higher binding affinity^35^. Based on these prior observations and the increased entropic penalty we measured by ITC, we hypothesized that the lower binding affinity for the *E. muelleri* complex reflects a lack of helical propensity for the individual peptides. Circular dichroism measurements (Fig. 5e) confirm that the EmMBD2/3 and EmGATAD2A/B peptides show relatively low helical content in isolation (5% and 19%, respectively) as compared to HsMBD2 and HsGATAD2A (25% and 66%, respectively)^35^. This observation further explains why the cross-species interactions bind with intermediate affinity. Replacing one of the interacting partners with the human ortholog with higher intrinsic helical propensity improves binding affinity proportionately (HsGATAD2A > HsMBD2). Finally, the DmMBD2/3 and DmGATAD2A/B coiled-coil domains show a higher level of conservation with the human orthologs (Fig. 5f). Binding analysis by ITC (Fig. 5d) confirms that DmMBD2/3 and DmGATAD2A/B form a stable heterodimeric complex with an affinity (K_D_ = 7,400 ± 1600 nM) that is over 100-fold weaker than the human orthologs.

**Figure 6:**
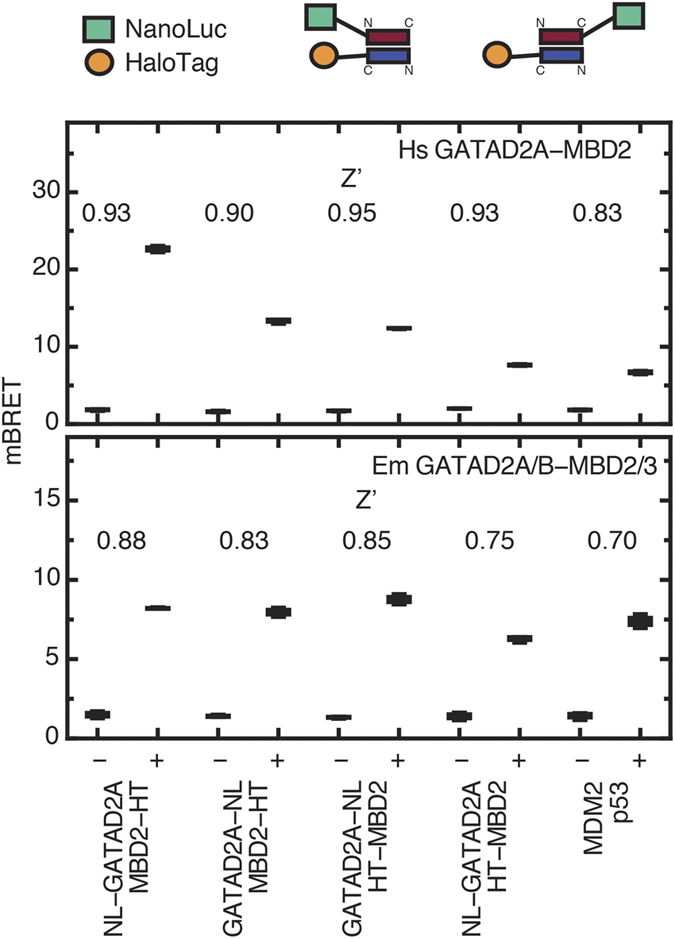
BRET analysis of coiled-coil complex formation. Measured BRET ratios are shown in boxplots for different donor and acceptor combinations of Hs (upper panel) and Em (lower panel) coiled-coil domains as well as the MDM2:p53 positive control. Each experiment was performed in triplicate with (+) and without (−) addition of the NanoBRET™ 618 fluorescent ligand and Z’ calculated and shown for each.

Finally, given the relatively weak binding affinity, we sought to determine whether the EmMBD2/3 and EmGATAD2A/B coiled-coil domains form a stable complex in cells. The NanoBRET™ bioluminescent resonance energy transfer assay^36^ uses a modified bioluminescent protein domain from *Oplophorus gracilirostris* (NanoLuc®)^37^ as the donor and the NanoBRET™ 618 fluorescent ligand that covalently attaches to a HaloTag® domain as the acceptor. We cloned EmGATAD2A/B and HsGATAD2A coiled-coil domains with both N- and C-terminal donor tags and the EmMDB2/3 and HsMBD2 coiled-coil domains with both N- and C-terminal acceptor tags. In Fig. 6, the measured BRET ratios are plotted for each of the four possible combinations with the calculated Z’ factor indicated. We observe efficient BRET for all possible combinations of both the Hs and Em coiled-coil domains, ranging from 6–9 mBRET for the *Ephydatia* complex, from 7–23 mBRET for the human complex, and 6–8 mBRET for the MDM2:p53 positive control. As would be expected for an anti-parallel coiled-coil complex, the measured BRET tends to be higher when the donor and acceptor domains are on opposite ends of the two peptides (N- and C-termini). These data are consistent with stable formation of the EmMBD2/3:EmGATAD2A/B coiled-coil complex in cells.

### Invertebrate coiled-coil domains have lower helical propensity

These data raise the question of why the binding affinity is so much higher in the human orthologs. Vertebrate genomes contain multiple MBD2 and GATAD2A paralogs, which could compete for the same binding partners. We previously demonstrated a hierarchy of binding affinity for GATAD2A between the human MBD2 paralogs (MBD2 ≈ MBD3 > MBD3L1 ≈ MBD3L2). The affinity differences reflect the relative helical propensity of the different coiled-coil domains^35^. Hence we hypothesize that competing paralogs in vertebrate genomes drive evolution of high affinity association between MBD2 and GATAD2A. Invertebrate species, on the other hand, have only one paralog of MBD2/3 and GATAD2A/B and, as a result, lack selective pressure for high affinity association. Note that EmMBD2a and EmMBD2b proteins in Fig. 1 represent two splice variants from the same gene, not distinct paralogs. This hypothesis predicts that invertebrate MBD2/3 and GATAD2A/B coiled-coil domains should have on average lower binding affinity and lower helical propensity.

**Figure 7:**
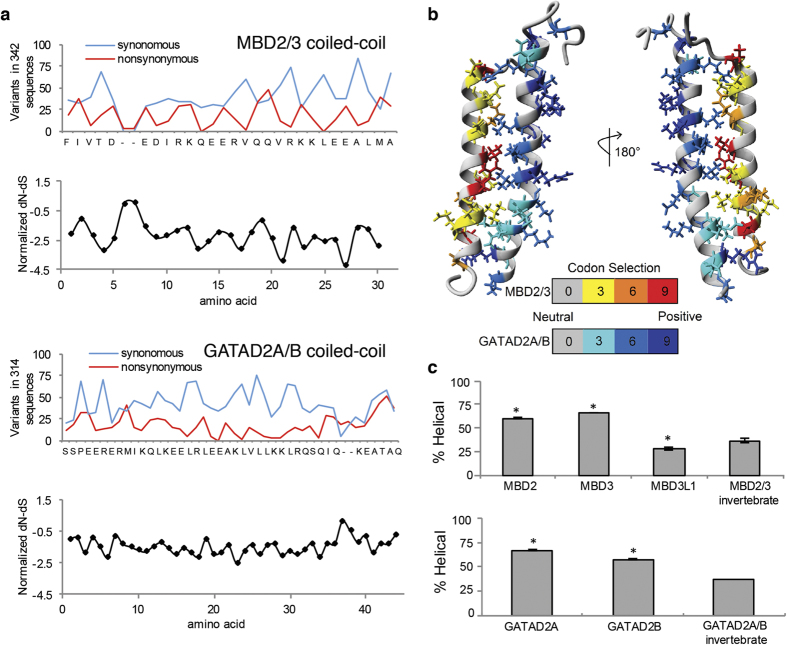
Conservation of coiled-coil interaction. (**a**) Synonymous and non-synonyous codon variation across all MBD2/3 and GATAD2A/B coiled-coil domains are plotted against the human sequences. (**b**) Codon selection is mapped onto the solution structure of the MBD2:GATAD2A coiled coil complex. Conserved residues are shown as sticks and color coded for codon selection. (**c**) Average helical propensity for the coiled-coil domains, as determined by the Agadir algorithm, is plotted for vertebrate and invertebrate MBD2/3 and GATAD2A/B orthologs.

To test this hypothesis, we compared helical propensity for MBD2 and GATAD2A paralogs across both vertebrate and invertebrate species. We first identified open reading frames for *MBD2* (103 sequences), *MBD3* (130 sequences), *MBD3L1–2* (48 sequences), *GATAD2A* (133 sequences) and *GATAD2B* (134 sequences) in vertebrate species using the NCBI database. These sequences were used to identify all non-redundant homologous sequences from invertebrate genomes in *MBD2/3* (46 sequences) and *GATAD2A/B* (47 sequences). Molecular phylogenetic analyses by the Maximum Likelihood method for *MBD2/3* (350 nucleotide sequences) and *GATAD2A/B* (314 nucleotide sequences) are shown in Supplementary Fig. S3. Synonymous and non-synonymous codon variations were calculated across each gene and plotted against the human sequences in Fig. 7a. Both domains demonstrate a fluctuating pattern of codon variation consistent with selective conservation of residues on one surface of the helices, i.e. every 3 to 4 amino acids. Mapping codon selection onto the structure of the complex shows selective conservation of residues at the protein-protein interface (Fig. 7b), consistent with preservation of the coiled-coil interaction. Interestingly, codon selection shows conservation of charged residues on the exposed surface of GATAD2A, which suggests this surface may be involved in a second interaction with another protein or DNA. Unlike the methyl-cytosine binding domain that shows genetic variation that interfere with DNA binding in invertebrates (i.e. *D. melanogaster*), all species analyzed to date, including all invertebrates, possess a functional coiled-coil domain. This suggests that even though MBD2/3 may have lost the ability to recognize and bind methylated DNA in some species, recruitment of GATAD2A/B has been maintained throughout evolution of the metazoa and this recruitment could be the primary function of MBD2/3 in species that lack cytosine methylation.

Next, the helical propensity for each homologous coiled-coil domains was calculated with the Agadir algorithm^38^,^39^ at pH 7, 278 K, and ionic strength of 0.1 M. Comparing the average helical propensity for the coiled-coil domain shows that vertebrate MBD2 and MBD3 have much higher helical propensity than MBD3L1 or the invertebrate MBD2/3 (Fig. 7c). Likewise, vertebrate GATADA2A and GATAD2B are significantly more helical than the invertebrate GATAD2A/B. These data support the hypothesis that the invertebrate coiled-coil complex binds with less affinity than the vertebrate orthologs.

### EmMBD2 function

**Figure 8:**
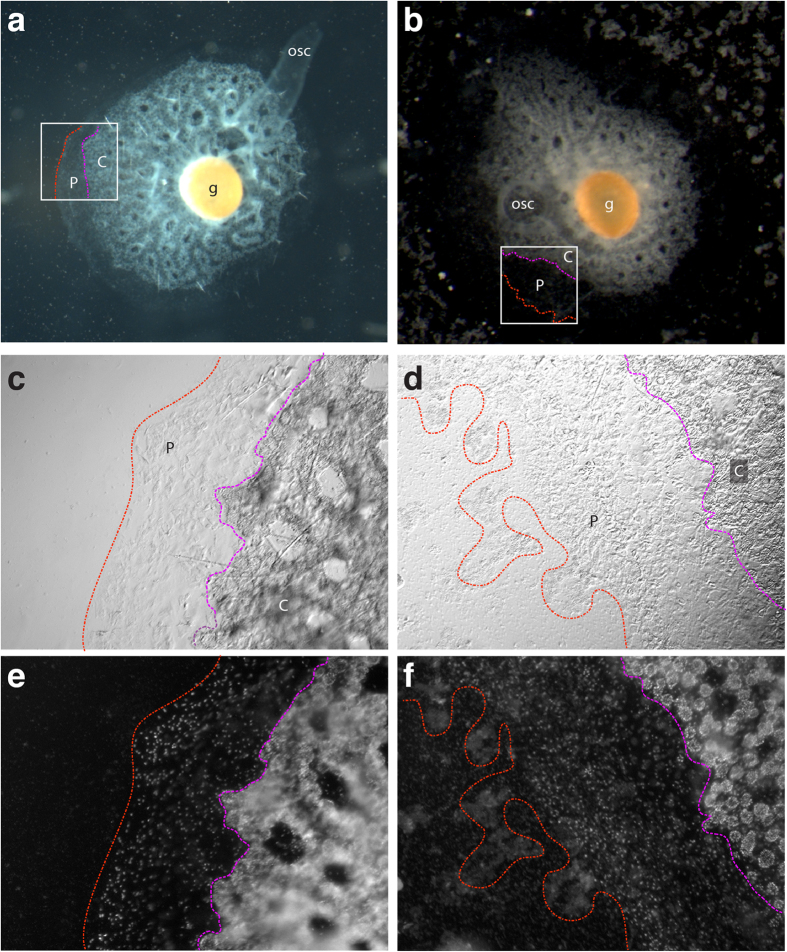
*E. muelleri* phenotypes following knockdown of EmMBD2. (**a** and **b**) Control and EmMBD2/3 RNAi-treated sponges photographed from above on stereomicroscope. (**a**) Control sponges were grown in Strekal’s media alone or soaked in dsRNA to GFP. (**b**) Sponges treated with dsRNA to EmMBD2/3. Leading edge of growth of basal pinacoderm (P); developing choanoderm nearest to basal pinacoderm (C), oscula (osc) and gemmule (**g**) are shown for reference. (**c**–**f**) Whole mount control and EmMBD2/3 RNAi-treated sponges. Magnified region as depicted in white boxes in A and B. Leading edge of basal pinacodermal (P) growth to left of images and between the red and pink lines. Developing choanoderm (C) is to right of pink line. (**c**) Control sponges show clearly delineated basal pinacoderm and proximal choanoderm development. (**d**) RNAi treated sponges show disorganized pinacoderm and proximal choanoderm. (**e**,**f**) Dapi staining of exact images shown in (**c** and **d**). Control and treated sponges show lack of connections (e.g., proper canals) forming between choanocyte chambers of the proximal choanoderm. Scale 1mm (**a** and **b**) and 200 μm (**c**–**f**).

Having established that EmMBD2/3 selectively binds methylated DNA and can recruit key components of NuRD, we sought to determine whether EmMBD2 functions during early sponge development and morphogenesis. We first tested if MBD2/3 is expressed during *E. muelleri* hatching and subsequent development to juvenile adult stage. We found expression across all developmental stages (Supplementary Fig. S4). We then treated sponges hatched from gemmules with dsRNA directed at EmMBD2/3 over the course of 72 hours. The average reduced expression after RNAi reduced expression of EmMBD2/3 across all experiments was 34%, which is within the expect range of 30–50%^24^. Compared to controls (Fig. 8a,c), RNAi treated sponges consistently show abnormalities along the leading edge of growth and within the pinacoderm (Fig. 8 and Supplementary Fig. S5). Whereas control sponges form a distinct basal pinacoderm layer (P) and proximally defined choanoderm (C) (Fig. 8c,e), treated sponges have a disorganized basal pinacoderm layer (Fig. 8d,f) and a less organized choanoderm (Fig. 8d,f region to right of pink line, and Supplementary Fig. S6). The RNAi treated sponges, however, do form oscula, canals, and choanocyte chambers and are able to filter water (Fig. 8b). Close inspection of the leading edge of the basal pinacoderm and developing choanoderm shows that the choanocyte chambers of the developing choanoderm do not organize with the clear formation of canals at the region nearest to the pinacodoerm (Compare Fig. 8c,e to d,f in the region to the right of pick line labeled C; also see Supplementary Fig. S6). These data support a hypothesis that DNA methylation may have a conserved regulatory role in developmental processes across metazoan evolution. Despite a change in the global methylation pattern, the MBD2-NuRD complex can recognize methylated DNA and may contribute to gene silencing even in the most ancient metazoan lineages still in existence.

## Conclusions

The studies presented here support a model in which the methylation-selective MBD2-NuRD complex evolved prior to the common ancestor for all metazoans. The methyl-cytosine binding domain of EmMBD2/3 preferentially binds methylated CpG dinucleotides in a manner that is biochemically and structurally very similar to vertebrate MBD2. Indeed, the atomic details of DNA binding lead to nearly identical NMR chemical shift dependencies despite over 600 million years of divergent evolution. Furthermore, the critical coiled-coil interaction between MBD2/3 and GATAD2A/B has been maintained across the same evolutionary distance. The binding affinity of this coiled-coil interaction, however, increases several orders of magnitude in vertebrate organisms. This change in affinity correlates with duplication of MBD2/3 and GATAD2A/B proteins, which leads us to hypothesize that competition among the various paralogs drives the evolution of high affinity association. Without this competition, micromolar binding affinity is sufficient for MBD2/3 to recruit a stable NuRD complex.

In addition, the presence of a functional and methylation-selective MBD correlates with the presence of DNA methylation. *D. melanogaster* does not methylate CpG dinucleotides to an appreciable extent and the MBD of DmMBD2 neither adopts a regular fold nor binds DNA. In contrast, coiled-coil domains of MBD2/3 and GATAD2A/B can be identified independent of the presence of DNA methylation. The latter suggests that the ability of MBD2/3 to bind and recruit NuRD has been retained throughout the animal kingdom while the DNA binding function was lost in some lineages. Finally, decreasing levels of of EmMBD2/3 expression by one-third of normal levels during development is sufficient to disrupt cellular organization of the developing sponge. This suggests that MBD2/3 has both methylation-dependent and independent functional roles that emerged with the earliest multicellular animals and have been maintained to varying degrees across animal lineages.

## Methods

### Identification of NuRD genes in E. muelleri

The *E. muelleri* transcriptome sequence was made available by Sally Leys (University of Alberta). *H. sapiens* gene sequences for each of the NuRD components and DNA methyl transferase enzymes (NCBI) were used as bait sequences for searching *A. queenslandica* genome (via BLAST® search on www.metazome.net/amphimedon) and *E. muelleri* transcriptome (via CLC Main Workbench V 6.8.2, www.clcbio.com). When necessary, orthologs from other invertebrate species (i.e. *C. elegans, D. melanogaster*, and *N. vectensis*) were first identified and subsequently used as bait sequences for searching the sponge sequences. To confirm gene identity, significant hits were searched against all non-redundant GenBank protein coding sequences (NCBI). Finally, conserved domains were identified by alignment with the *H. sapiens* orthologs and the Conserved Domain Database at NCBI.

### DNA methylation and expression of EmMBD2/3 in E. muelleri

Genomic DNA was extracted from *E. muelleri* tissues at three developmental time points: Stages 0–1 (multipotent stem cells migrating out of gemmule to establish connection with substrate), Stages 2–4 (sponge body plan forming), and Stage 5 (juvenile sponge with fully developed aquiferous system) using a modified CTAB extraction protocol^40^. For each of the three developmental stage ranges, approximately ten sponges were pooled for the DNA isolations in each trial. We performed three independent trials. DNA methylation of *E. muelleri* developmental stages was quantified using the MethylFlash™ Methylated DNA Quantification Kit (Colorimetric), according to manufacturer protocol (Epigentek Inc.). Two hundred nanograms of DNA per sample were analyzed in each trial. Absorbance at 450 nm was assayed using a Beckman Coulter DTX880 microplate reader.

Genomic DNA isolated by modified CTAB^40^, treated with RNase A and purified by chloroform extractions from *E. muelleri* tissues at Stage 0–1, Stages 2–4, and Stage 5 (as described above) was sent to Zymo Research (Irvine, CA) for SRM-based mass spectrometry analysis to quantify 5-methy-2′-deoxycytidine (5mdC) and 5-hydroxymethyl-2′-deoxycytidine (5HmdC). 5HmdC and 5mdC concentrations were measured as a percentage of 2′-deoxyguanosine (dG) (e.g. [5HmdC]/[dG] and [5mdC]/[dG]). The calibrated ranges for the analytes were 0–2.5% for 5HmdC and 0–25% for 5mdC using a fixed 40 pmol amount of dG as an internal standard. Values below the estimated limit of quantification of 0.01% are not considered to be reliable.

RNA was isolated from *E. muelleri* sponge tissue harvested at developmental stages or after RNAi treatment using the RNeasy® Mini Kit (Qiagen), limiting genomic DNA contamination through an additional on-column DNase I treatment. Equal amounts of cDNA (150–200 ng/ul) were synthesized from sponge mRNA using Superscript® VILO reverse transcriptase master mix (ThermoFisher Scientific). SYBR Green (ThermoFisher Scientific) chemistry and Chromo4 (BioRad Laboratories) were used for qRT-PCR with gene specific primers (F: 5′-TCCGACATTGCGTTCCACAG-3′; R: 5′-AGCGTTGGTAGATCGTGGAG-3′). EmMBD2/3 expression levels were normalized to the housekeeping gene *Ef1a* and expression was quantitated as described in ref. 41.

### Protein and DNA purification

The MBD of HsMBD2a (amino acids 150–214) was expressed and purified as described previously^13^. In a similar manner, the MBD (amino acids 16–83) and coiled-coil domain (amino acids 324–354) of EmMBD2/3a, the coiled-coil domain (amino acids 58–100) of EmGATAD2A/B, the MBD (amino acids 1–87) and coiled-coil domain of DmMBD2/3 (amino acids 309–339), and the coiled-coil domain of DmGATAD2A/B (amino acids 183–223) were cloned into a modified pET32a vector^42^ as C-terminal fusions to bacterial thioredoxin with an intervening thrombin cleavage site. The resulting vectors were transformed in Rosetta™2(DE3) (Novagen) cells, grown in Luria Broth medium to an optical density at 600 nm of ~0.8, induced with 1 mM isopropyl β-D-1-thiogalactopyranoside, and harvested after 2 hours. The bacterial pellets from 1 L of culture were re-suspended in 20 mL of B-PER bacterial protein extraction reagent (Thermo Fisher) supplemented with 0.5 M NaCl and lysed by sonication. The soluble lysate was passed over a nickel column and protein eluted with 300 mM imidazole. Purified protein was dialyzed overnight against 20 mM Tris pH 8.0, 150 mM NaCl, 2 mM β-mercaptoethanol. For circular dichroism and NMR studies, the thioredoxin fusion tag was removed by the addition of 100 U of thrombin; for binding studies and analytical ultracentrifugation analyses the thioredoxin tag was not removed. The proteins were further purified by ion exchange chromatography over Source 15S (DmMBD2/3 MBD, EmMBD2/3 MBD, and GATAD2A/B coiled-coil domains) or Source 15Q (EmMBD2/3, DmMBD2/3, and DmGATAD2A/B coiled-coil domains) 4.6/100 columns (GE Healthcare) followed by gel filtration over a Superdex75 26/60 column (GE Healthcare). Proteins were exchanged into appropriate experimental buffer and final concentrations determined by UV absorption at 280 nm.

Seventeen base pair oligonucleotides (forward: GAGGCGCT(***m**)**CG***GCGGCAG; reverse: CTGCCGC(***m**)**CG***AGCGCCTC) with and without methylation of the central CpG dinucleotide (highlighted in italics and bold) were purchased (IDT), annealed, and purified by ion exchange chromatography over a Source 15Q 4.6/100 column (GE Healthcare). Final DNA concentration was determined by UV absorption at 260 nm.

### Molecular dynamics simulations

Initial structures of the EmMBD2/3 MBD:DNA and EmMBD2/3:GATAD2A/B coiled-coil complexes were generated from the solution structure of *G. gallus* MBD2:DNA^12^ and human MBD2:GATAD2A coiled-coil^34^ complexes, respectively, by comparative modelling using the MODELLER program^30^. The methylated DNA sequence was generated with the 3D-DART webserver and subsequent molecular dynamics simulations were performed essentially as described previously^15^ using NAMD 2.9^43^ and CHARMM27 force field^44^. In brief, the initial complexes were solvated in a box with at least 10 Å of surrounding TIP3P water and 75 mM NaCl and equilibrated with two rounds of NVT simulations (5000 steps of minimization followed 30 ps of dynamics at 300 K) with rigid (first round) or 5 kcal/mol/Å harmonic (second round) restraints. Finally, a preparative 1 ns NPT simulation at 300 K was followed by 40 ns of unrestrained NPT dynamics.

### NMR spectroscopy

Purified protein was added to 10% excess DNA, buffer exchanged into 10 mM NaPO4, pH 6.5, 1 mM dithiothreitol, 10% ^2^H_2_O and 0.02% sodium azide and concentrated to 0.2–0.5 mM. 2D ^15^N-HSQC spectra and 3D HNCO, HNCA, HNCACB, CBCACONH, ^15^N-NOESY-HSQC were collected on a Bruker Avance III 700 MHz instrument at 25 °C and data were processed and analyzed with NMRPipe^45^ and CcpNmr^46^. The backbone resonances were assigned using standard procedures and have been deposited to the Biological Magnetic Resonance Bank (BMRB ID: 26931).

### Surface plasmon resonance

DNA binding affinity was determined by surface plasmon resonance on a Biacore T100 (GE Healthcare) using steady state analysis as described previously^12^,^14^. Briefly, 3′ biotinylated 17 base pair oligonucleotides were bound to a Sensor SA chip (10 ng/ul DNA, 10 ul/min flow rate) until a relative response of approximately 50 RU. Protein was buffer exchanged in SPR binding buffer (10 mM HEPES, 50 mM NaCl, 3 mM MgCl2, 0.1 mM EDTA, 1 mM DTT, 0.05% polysorbate 20, pH 7.4) and binding response measured at a flow rate of 30 μL/min. The steady state response was measured for a series of protein concentrations ranging from 1 μM to 2.5 nM or 100 μM to 250 nM for binding to methylated and unmethylated DNA, respectively. A single concentration from the middle of the titration was repeated three times to estimate error in measurement. The steady state response was first normalized to that of the immobilized DNA such that the maximum response (R_max_) reflects the stoichiometry of binding (as previously described^14^,^47^) and the data fit to a one-site binding model with pro Fit software (QuantumSoft).

### Isothermal titration calorimetry

The fusion peptides were left uncleaved, purified, and buffer exchanged into 50 mM Hepes pH 7.4 and 150 mM NaCl. Measurements were performed on a MicroCal Auto-iTC200 calorimeter (GE Healthcare). HsMBD2 (1.8 mM), EmGATAD2A/B (2.74 mM), and DmGATAD2A/B (2.5 mM) coiled-coil domains were injected into HsGATAD2A (100 μM), EmMBD2/3 (270 μM) and DmMBD2/3 (250 μM) coiled-coil domains, respectively, for a total of 20–24 injections at a temperature of 298 K. The resulting isotherms were fit to a one-site binding model using Origin 7.0 software as described previously^35^.

### Circular dichroism and analytical ultracentrifugation

For circular dichroism studies, the coiled-coil domain bacterial expression constructs were mutated to introduce an N-terminal tyrosine for accurate concentration determination of the isolated peptides by UV spectroscopy and the thioredoxin tag removed by thrombin cleavage during purification. The purified proteins were buffer exchanged (10 mM sodium phosphate, pH 6.5) and concentrated to 50 μM. Circular dichroism spectra were collected on a Chirascan™-Plus CD Spectrometer (AppliedPhotophysics) at 293 K, scanning from 185–269 nm, 0.5 nm intervals, and 24 nm/min. The resulting spectra were normalized to give molar ellipticity and the helical content calculated based on ellipticity at 222 nm^48^.

For analytical ultracentrifugation, the fusion proteins were left uncleaved but otherwise purification was performed as described. Sedimentation velocity was measured at 40,000 rpm, 293 K, under physiological buffer conditions (20 mM Tris pH 8.0, 150 mM NaCl) on a Beckman Optima XL-I analytical ultracentrifuge (Beckman Coulter Inc.) equipped with a four and eight-position AN-60Ti rotor. Data were analyzed with the SEDNTERP^49^ and SEDFIT^50^ programs as described previously^35^.

### Bioluminescent resonance energy transfer

The coiled-coil domains of HsGATAD2A (amino acids 138–178) and EmGATAD2A/B (amino acids 58–100) were cloned into the pNLF1-N and pNLF1-C mammalian expression vectors (Promega) to generate N- and C-terminal NanoLuc® fusions. The coiled-coil domains of HsMBD2 (amino acids 360–392) and EmMBD2/3a (amino acids 324–354) were cloned into the pHTN and pHTN mammalian expression vectors (Promega) to generate N- and C- terminal HaloTag® fusions. HEK293T cells were co-transfected with NanoLuc® and HaloTag® fusion pairs (1:10 ratio, respectively) with FuGene® (Promega) according to the manufacturers instructions. Cells were incubated (37 °C, 5% CO_2_) for 16–20 hours before splitting into 96-well plates. For each HaloTag® and NanoLuc® fusion pair, cells were split into two pools and mixed with either 100 nM HaloTag® Ligand 618 or 0.1% DMSO control. Each pool was plated in triplicate and incubated 18–24 hours. Nano-Glo® substrate was added to each well to a final concentration of 1x per the manufacturers instructions. The plate was rocked for 30 seconds to mix and then read immediately with a ClarioStar plate reader. NanoLuc® bioluminescence was detected at 460 nm and HaloTag® emission was read at 618 nm. Raw values were converted to milliBRET units (mBU) by taking 1,000 times the ratio of 618 nm emission to 460 nm emission. Reliability of the assay was assessed by calculating the Z’ factor,


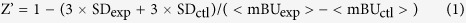


where SD is the standard deviation and <mBU> is the mean milliBRET of the experimental (exp) and no ligand control (ctl).

### Phylogenetic analysis of coiled-coil domains

Open reading frames for vertebrate *MBD2, MBD3, MBD3L1–5, GATAD2A*, and *GATAD2B* were obtained from NCBI Gene. These sequences were used to identify orthologs in *C. elegans, D. melanogaster*, and *A. mellifera* by BLAST® search, the results of which were then used to identify all non-redundant nucleotide invertebrate sequences with homology. The identified sequences were aligned with ClustalW codon. Phylogenetic trees were generated using Maximum Likelihood method based on the Tamura-Nei model^51^ with 5000 bootstrap replicates^52^ using MEGA5^53^. Codon selection analyses were performed by HyPhy with the Tamura-Nei model followed by calculation of the z-score of dN-dS for each amino acid. Z-scores greater than one were scored 9, those greater than 0.5 scored 6, and those greater than 0 scored 3. The helical propensity of each sequence obtained above for the coiled-coil domain was predicted by Agadir^54^ at pH 7, 278 K, and ionic strength of 0.1.

### Functional analysis of EmMBD2/3

To reduce expression of *EmMBD2/3*, RNAi was used to knockdown target gene expression in *E. muelleri* tissues as previously described^24^, with some modifications. dsRNA was synthesized to target suppression of *EmMBD2/3* using the T7 RiboMAX^TM^ Express RNAi *in vitro* transcription system (Promega). Sponges were treated by soaking with 10 ng/μL dsRNA in 1X Strekal’s medium over the course of 48–72 hours. Media and dsRNA was replaced daily. Control sponges were either grown in Strekal’s media or in Strekal’s with 10 ng/μL dsRNA to GFP. Levels of gene expression were assayed post-RNAi treatment using qRT-PCR as described^24^,^41^.

After RNAi treatment, sponges were washed several times in Strekal’s media and photographed on an Olympus SZX12 stereomicroscope and then either harvested for RNA or DAPI stained and mounted for visualization on an Olympus BX61 microscope.

## Additional Information

**How to cite this article**: Cramer, J. M. *et al*. Methylation specific targeting of a chromatin remodeling complex from sponges to humans. *Sci. Rep.* **7**, 40674; doi: 10.1038/srep40674 (2017).

**Publisher's note:** Springer Nature remains neutral with regard to jurisdictional claims in published maps and institutional affiliations.

## Supplementary Material

Supplementary Information

## Figures and Tables

**Table 1:** DNA methylation in *E. muelleri*.

Experiments	Stage 0–1			Stage 2–4			Stage 5		
Trial	1	2	3	1	2	3	1	2	3
5-mC/Total DNA (ELISA)*	0.215	**0.430**	0.465	0.205	**0.408**	0.701	0.405	**0.769**	1.247
%5mdC (SRM-based MS)*	5.056	**5.145**	5.394	4.570	**4.620**	4.739	4.935	**4.427**	4.377
%5HmdC (SRM-based MS)*	0.011	**0.017**	0.018	**0.008**	0.007	0.009	nd**	nd	nd

^*^Median value for the three replicates is highlighted in bold.

^**^nd – not detected.

**Table 2:** Binding affinity for DNA.

Protein	DNA	Kd (μM) ± SE*	R_max_ ± SE**
HsMBD2	mCpG	0.33 ± 0.02	0.93 ± 0.01
HsMBD2	CpG(x3)	81.7 ± 0.5	6.0 ± 0.1
EmMBD2	mCpG	0.43 ± 0.01	0.96 ± 0.01
EmMBD2	CpG (x3)	65.0 ± 0.7	6.5 ± 0.1

^*^SE – standard error.

^**^Normalized such that Rmax reflects stoichiometry (n).

**Table 3:** Binding affinity of coiled-coil complexes.

Complex	K_D_ (nM)*	n	∆H (cal/mol)*	−T∆S (cal/mol)	∆G (cal/mol)
HsMBD2:HsGATAD2A	48 ± 16	1.3	−9,930 ± 50	60	−9,990
EmMBD2/3:HsGATAD2A	380 ± 30	1.3	−8,990 ± 40	240	−8,750
HsMBD2:EmGATAD2A/B	1,300 ± 100	1.1	−8,920 ± 60	900	−8,020
EmMBD2/3:EmGATAD2A/B	9,200 ± 700	0.8	−9,800 ± 100	2,900	−6,900
DmMBD2/3:DmGATAD2A/B	7,400 ± 1600	1.0	−27,600 ± 800	20,500	−7,100

^*^Standard deviation is reported for fit values (K_D_ and ∆H).
