# Supplementary material for: Methylation specific targeting of a chromatin remodeling complex from sponges to humans
Source: Sci Rep. 2017 Jan 17;7:40674. doi: 10.1038/srep40674 (PMC5240623; doi:10.1038/srep40674)
Supplement: Supplementary Information [file srep40674-s1.pdf]

Supplemental Information

Methylation specific targeting of a chromatin remodeling complex from sponges to humans

Jason M. Cramer[1,†], Deborah Pohlmann[2,†], Fernando Gomez[2], Leslie Mark[2], Benjamin Kornegay[2], Chelsea Hall[2], Edhriz Siraliev-Perez[3], Ninad M. Walavalkar[4,†], M. Jeannette Sperlazza[5], Stephanie Bilinovich[4], Jeremy W. Prokop[6], April L. Hill[2], and David C. Williams Jr[4,*]

Supplementary Figure S1. RMSF for molecular dynamics simulations. The RMSF calculated over a sliding window of 10 steps is plotted for the (a) EmMBD2/3-DNA and (b) EmMBD2/3-GATAD2A/B molecular dynamics simulations. The calculated RMSF includes the well-structured regions of the individual complexes: (a) EmMBD2a residues 20-74 and DNA base pairs 3-14; (b) EmMBD2/3a residues 330-351 and GATAD2A/B residues 64-86).

Supplementary Figure S2. Gel filtration analysis of coiled-coil complex formation. The coiled-coil domains were purified as thioredoxin fusions and buffer exchanged into 20 mM Tris pH 8.0, 150 mM NaCl. The elution profile for gel filtration analysis on a Superdex 10/300 (GE Healthcare) is shown for 100 µM samples of EmGATAD2A/B (blue), EmMBD2/3 (red), and a 1:1 mixture (black).

Supplementary Figure S3. MBD2/3 and GATAD2A/B Sequences. Phylogenetic trees were created with Maximum Likelihood method based on the Tamura-Nei model using 1000 bootstrap replicates. Values at each node represent the percent of replicate trees from the bootstrap analysis that cluster together. A total of 350 (MBD2/3) and 314 (GATAD2A/B) nucleotide sequences were used for 1596 (MBD2/3) and 132 (GATAD2A/B) positions. Newick formatted files are provided as separate attachments**.**

Supplementary Figure S4. EmMBD2/3 Expression levels. Sponges were hatched from gemmules in Strekal's media and collected at the desired developmental stage (after Rivera et al, 2013) for RNA extraction. qRT-PCR was used to measure EmMBD2/3 expression and was normalized to the housekeeping gene EmEf1a expression.

Supplementary Figure S5. E. muelleri phenotypes following knockdown of EmMBD2 without line annotations. (a and b) Control and EmMBD2/3 RNAi-treated sponges photographed from above on stereomicroscope. (a) Control sponges were grown in Strekal's media alone or soaked in dsRNA to GFP. (b) Sponges treated with dsRNA to EmMBD2/3. Leading region of growth of basal pinacoderm denoted by arrowheads and oscula (osc) and gemmule (g) are shown for reference. (c – f) Whole mount control and EmMBD2/3 RNAi-treated sponges. Leading edge of basal pinacodermal (pin) growth on left of figure and developing choanoderm (cho) on right. (c) Control sponges show clearly delineated pinacoderm and choanoderm development. (d) RNAi treated sponges show disorganized pinacoderm and choanoderm. (e-f) Dapi staining of images shown in c and d. Control and treated sponges show lack of connections (e.g., proper canals) forming between choanocyte chambers (red arrows) of the proximal choanoderm. Scale 1mm (a and b) and 200μm (c-f).

Supplementary Figure S6. E. muelleri phenotypes following knockdown of EmMBD2. (a, c) Control sponges imaged by laser confocal microscopy in developing choanoderm proximal to basal pinacoderm. Lining of a canal delineated by dashed line. Representative choanocyte chambers lining canals are labeled with CC. (b, d) RNAi treated sponges imaged by lasar confocal microscopy in developing choanoderm proximal to basal pinacoderm. Canals and connections between choanocyte chambers are less organized when EmMBD2/3 is knocked down. Representative choanocyte chambers lining disorganized canals are labeled with CC. Scale 25 μm.

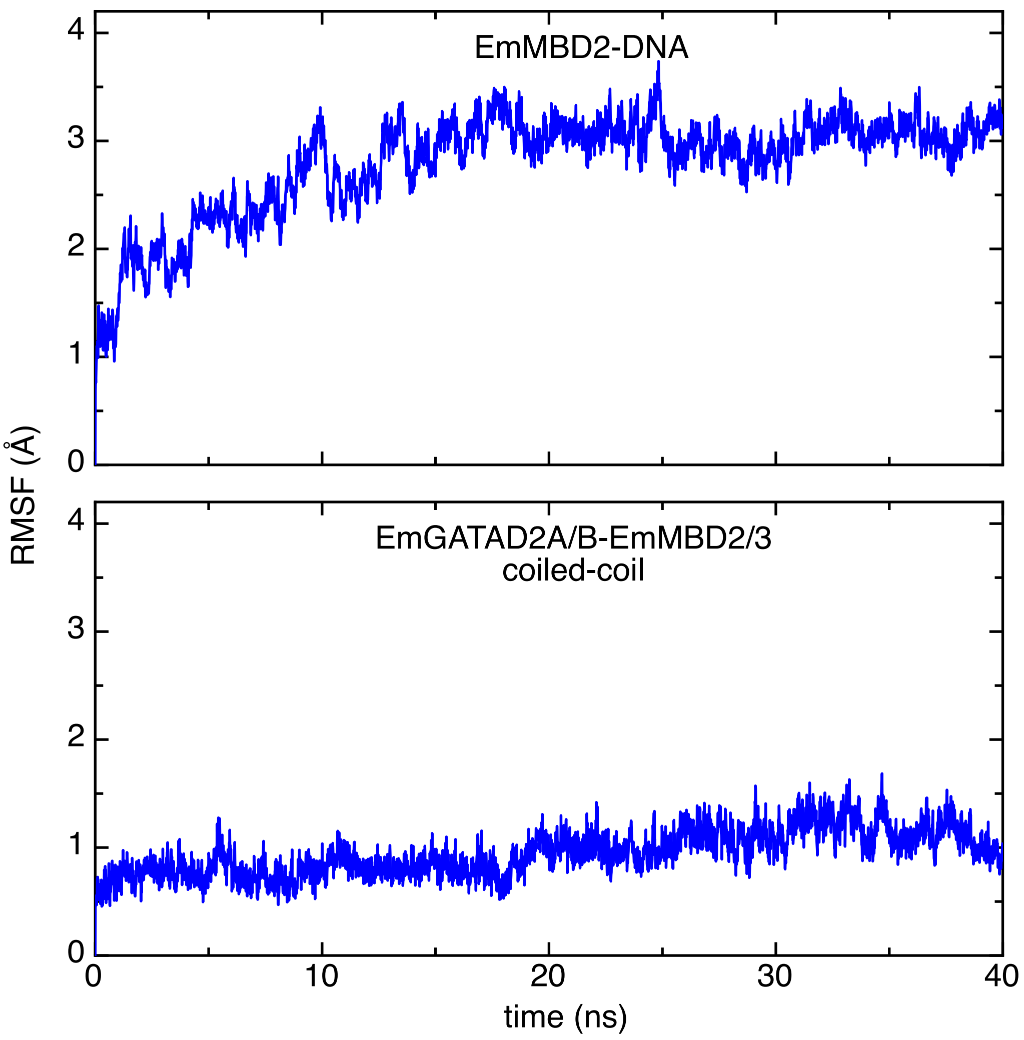

Supplementary Figure S1

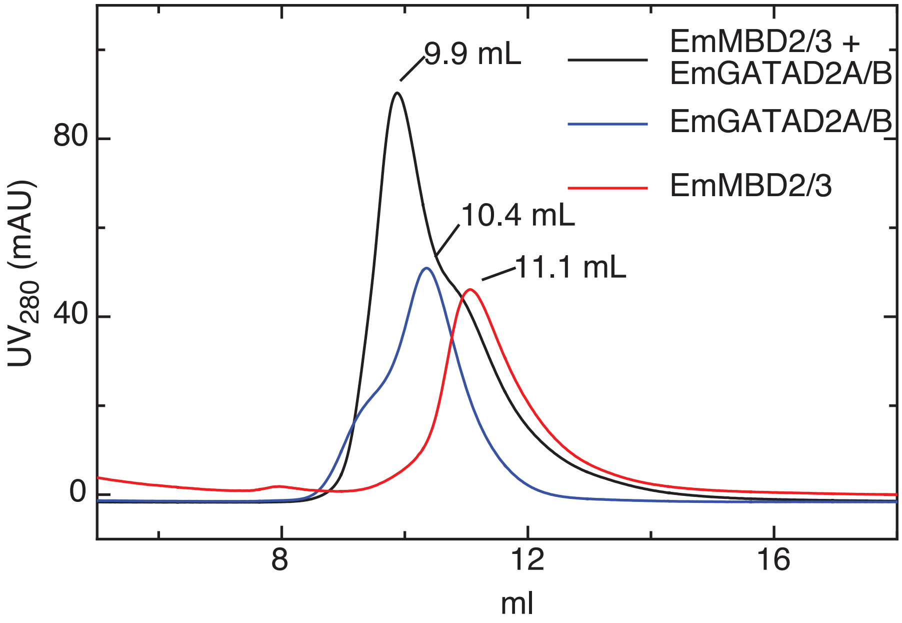

Supplementary Figure S2

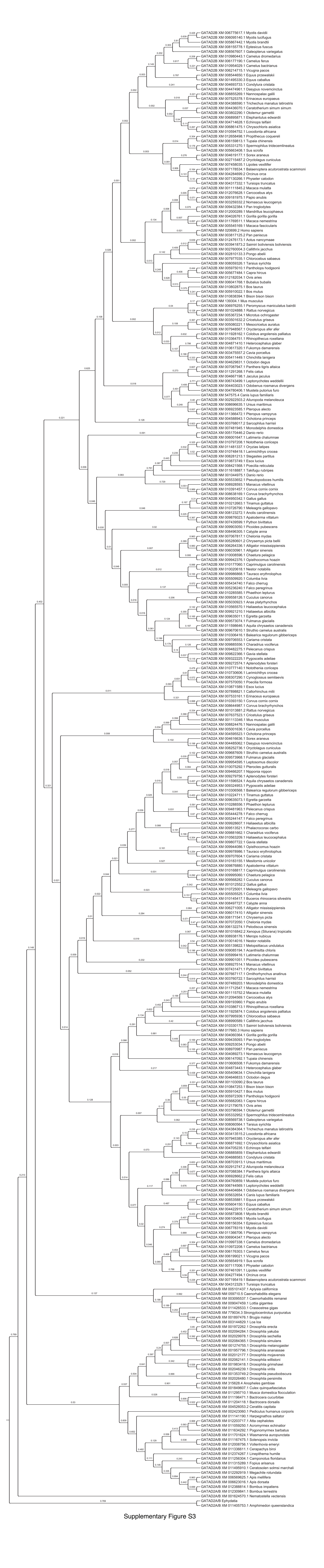

Supplementary Figure S3

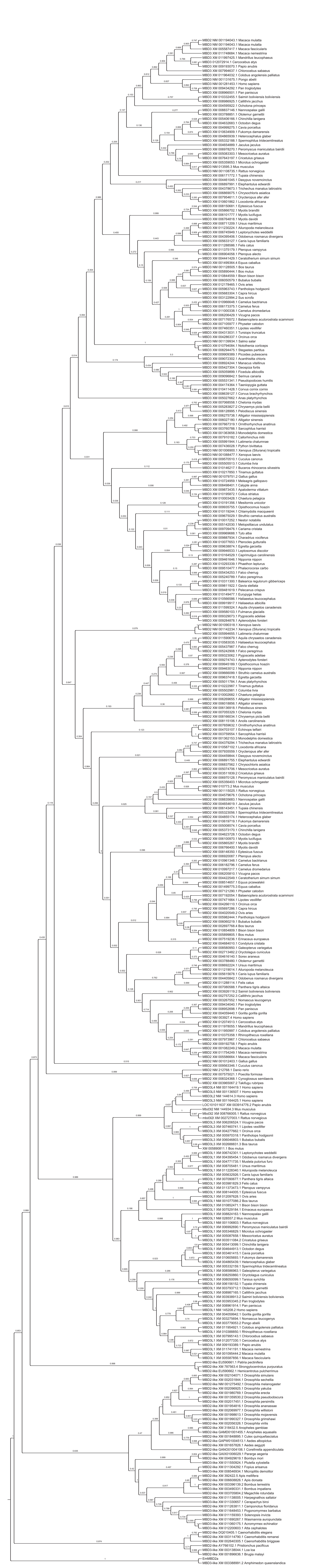

Supplementary Figure S3

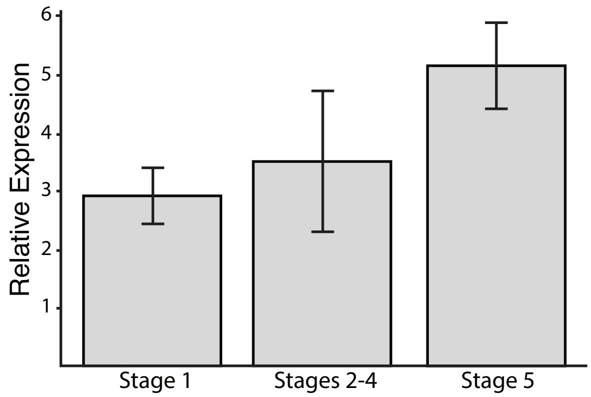

Supplementary Figure S4

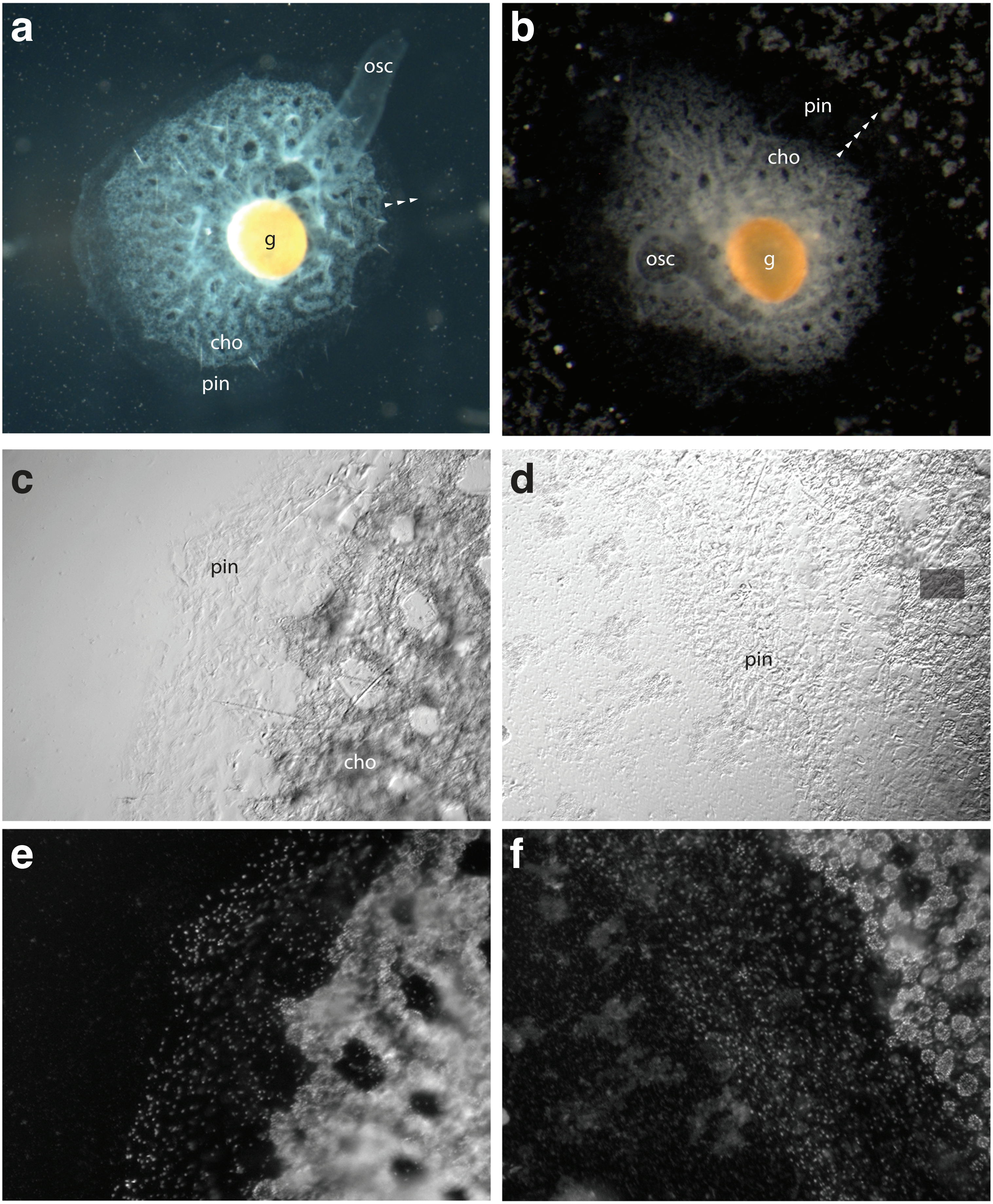

Supplementary Figure S5

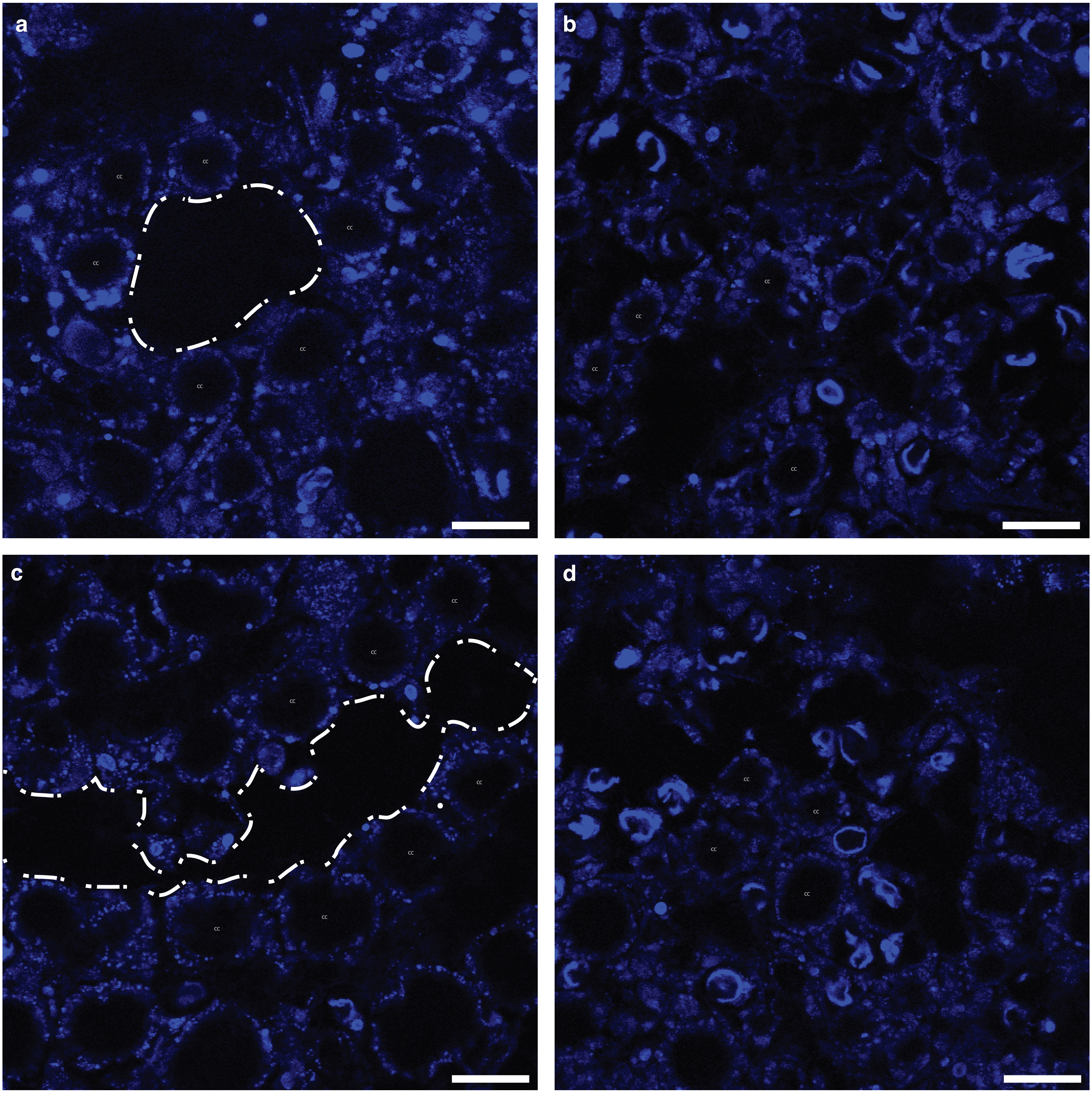

Supplementary Figure S6
